# T cell unresponsiveness in a pediatric cystic fibrosis patient: a case report

**DOI:** 10.1186/1710-1492-10-2

**Published:** 2014-01-17

**Authors:** Rahul Kushwah, Stéphane Gagnon, Neil B Sweezey

**Affiliations:** 1 McMaster Stem Cell and Cancer Research Institute (SCC-RI), Faculty of Health Sciences, McMaster University, Hamilton, ON, Canada; 2 Physiology and Experimental Medicine, Research Institute, The Hospital for Sick Children, Toronto, ON, Canada; 3 Respiratory Medicine, Physiology and Experimental Medicine, The Hospital for Sick Children, 555 University Avenue, Toronto, ON M5G 1X8, Canada

**Keywords:** T cell exhaustion, Cystic fibrosis, Naïve T cells, T cell differentiation

## Abstract

A girl was diagnosed with cystic fibrosis (CF) at birth, with repeatedly positive sweat tests and homozygous F508del mutations of her CF transmembrane conductance regulator *(CFTR)* gene. From an early age, her lung disease was more severe than her birth cohort peers despite aggressive treatment. At the age of 16 she was listed for lung transplantation, but prior to transplant was not on systemic corticosteroids or other immunosuppressive agents. In response to *ex vivo* stimulation, her pre-transplant peripheral blood T cells unexpectedly failed to produce detectable levels of IFN-γ, unlike cells from healthy controls or from another girl with CF and lung disease of comparable severity. Furthermore, naïve T cells freshly isolated from her peripheral blood showed a complete block of T cell differentiation into Th1, Th17 and Treg lineages, even in the presence of cytokines known to promote differentiation into the respective lineages. Her serology has been remarkably devoid of evidence of exposure to viruses that have been associated with T cell exhaustion. However, her freshly isolated naïve T cells showed sustained expression of markers of T cell exhaustion, which were further induced upon *ex vivo* stimulation, pointing to T cell exhaustion as the cause of the failure of naïve T cells to undergo differentiation in response to cytokine stimulation. Although excessive inflammation in CF lung can be both ineffective at clearing certain pathogens as well as destructive to the lung tissue itself, adequate inflammation is a component of an effective overall immune response to microbial pathogens. Our present findings suggest that intrinsic impairment of T cell differentiation may have contributed to the greater severity and more rapid progression of her CF lung disease than of the lung disease of most of her peers.

## Background

Cystic fibrosis (CF), an autosomal recessive disease caused by mutations in the CF transmembrane conductance regulator (*CFTR*) gene [1], is an inherited, life-limiting condition (reviewed in [2]). The main cause of death is CF lung disease [3], with a vicious cycle of infection and inflammation and interspersed acute exacerbations [2]. From early in life the typical CF patient, compared to healthy controls, has evidence of sustained and severe lung inflammation [4] and CF cells display a hyper-inflammatory phenotype that is ineffective at clearing bacterial pathogens but rather causes progressive tissue damage [5]. T lymphocytes predominate in the CF airway wall mucosa and submucosa, where the most severe tissue damage is noted in patients with the most advanced lung disease [6,7]. Studies have reported a skewing of CF T cell immune responses toward the Th2 and more recently Th17 lineages [8], with increased levels of pro-inflammatory cytokines of the Th17 family implicated in the destructive acute exacerbations [9]. In this context, we were surprised to discover that one of our patients with advanced CF lung disease, whilst on the list for lung transplantation, demonstrated evidence of T cell exhaustion with failure of naïve T cells to undergo differentiation into cytokine producing effector cells in response to stimulation. We speculate that an intrinsic, primary impairment of T cell differentiation may have contributed to the greater severity of her CF lung disease compared to her peers.

## Case presentation

### Clinical history

Born at term in southern Ontario, Canada, of non-consanguineous Caucasian parents following an uneventful pregnancy, the patient was appropriate in size for a full term baby and was discharged from hospital at four hours of age. She was initially breast fed but readmitted to hospital on the third day of life for failure to pass meconium. Intestinal perforation was noted and about 15 cm of bowel were resected. A diagnosis of CF was confirmed by repeated sweat chloride tests, consistent with her *CFTR* genotype of F508del homozygote. She has been followed regularly at the multidisciplinary CF Clinic of The Hospital for Sick Children, Toronto, on a quarterly basis at a minimum. Maintaining adequate weight gain has been a challenge. Although respiratory symptoms were not recognized in the immediate newborn period, at three months she had a severe pulmonary infection with Respiratory Syncitial Virus. By the age of 9 months, her chest X-Ray had early bronchiectatic changes that progressed to diffuse cystic bronchiectasis at 12 years. Her respiratory secretions intermittently grew *Pseudomonas aeruginosa* since the age of two years, *Staphylococcus aureus* since four years, as well as (less frequently) *Haemophilus influenzae* and *Stenotrophomonas maltophilia.* Although *Aspergillus fumigatus* has been identified several times, her serum IgE levels and skin tests for aspergillus allergy have been consistently normal, arguing against a diagnosis of allergic bronchopulmonary aspergillosis. From an early age, her pulmonary function has been more compromised than that of her birth cohort peers at our institution. Despite aggressive treatment of an increasingly frequent series of acute pulmonary exacerbations, her FEV_1_ during her 18th year was consistently below 30 and for several months she required 3 - 4 L/min of supplemental oxygen to maintain her hemoglobin oxygen saturations over 95%. Until her lung transplantation just before her 18th birthday, she had not been on systemic corticosteroids or other immunosuppressive agents. Her serology had been negative for IgG antibody to cytomegalovirus (CMV) by enzyme immunoassay (5 times out of 5 assessments over the last 15 months) and also negative when assayed (on at least one occasion) for IgG antibodies against each of Epstein-Barr virus early or nuclear antigens, hepatitis C virus, human immunodeficiency viruses 1 or 2, herpes simplex virus, or *Toxoplasma gondii*. Her most recent serology was also negative for IgG antibody to Epstein-Barr virus capsid antigen, although she had been positive for this antibody once over a year ago. No evidence was found for adenoviral DNA by polymerase chain reaction, or for hepatitis B surface antigen or HIV P24 antigen by chemiluminescence immunoassay. No antigens of the following respiratory viruses were detected on immunofluorescence microscopy of respiratory secretions on nasopharyngeal swabs: parainfluenza viruses 1, 2, 3, adenovirus, respiratory syncytial virus, human metapneumovirus, influenza viruses A, B. Nevertheless, we have evidence of good antibody responses to her vaccines against measles, mumps, rubella, varicella zoster virus and hepatitis B.

### Immunological analysis of T cell response

**Figure 1:**
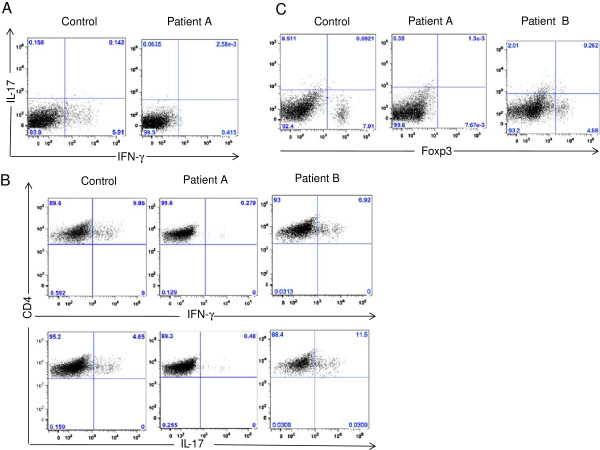
**CF patient A (the subject of this case report), but not CF patient B (another patient with CF lung disease of a similar degree of severity) had reduced T cell responses compared with control. (A)** IL-17 and IFN-γ production by T cells from mononuclear cells following overnight stimulation. T cells from patient A produced deficient IL-17 and IFN-γ compared to control. **(B)** Naïve T cells were isolated from patients A, B and a healthy control subject using magnetic sorting and differentiated into Th1 and Th17 lineages. Shown are representative flow cytometry plots of production from differentiated Th1 and Th17 cells, respectively. Patient A, but not patient B, produced reduced levels of IFN-γ and IL-17 compared with control. **(C)** Naïve T cells were differentiated into regulatory T cells. Shown are representative flow cytometry plots of Foxp3 expression. Patient A, but not patient B, produced reduced levels of Foxp3 compared with control. Representative of 2 - 3 independent experiments.

We carried out immunological analysis of T cell response in the peripheral blood of this patient prior to her lung transplantation. Peripheral blood mononuclear cells were isolated and stimulated with anti-CD3 and anti-CD28 antibodies to assess for cytokine production from T cells [10]. Whereas T cells amongst the mononuclear cells from healthy controls produced identifiable levels of IFN-γ which could be detected by flow cytometry [11], T cells from the patient failed to show any IFN-γ production (see Figure 1A). This could either be explained by massive immunosuppression within the immune system of the patient or an unlikely scenario in which her T cells are intrinsically defective in cytokine production. In order to test for this scenario, we isolated and carried out differentiation of naïve CD4+ T cells into inflammatory T helper type 1 (Th1) or T helper type 17 (Th17) lineages, or into regulatory T cells (Tregs), using exogenous cytokines [12,13]. Although T cells from healthy controls were able to differentiate into Th1 and Th17 lineages, detected by production of IFN-γ and Th17 respectively, along with differentiation into Tregs detected by expression of the transcription actor Foxp3 [14], T cells from the patient showed a complete block in T cell differentiation into the three T cell lineages tested [12,13], pointing towards an intrinsic loss of T cell responsiveness to differentiate in the presence of exogenous cytokines (Figures 1B, C). Furthermore, this block in T cell differentiation was not specific for one particular lineage but was spread across all three lineages. Loss of responsiveness to exogenous cytokines led us to hypothesize that the patient could be demonstrating T cell exhaustion.

### Nature of T cell exhaustion

T cell exhaustion is a condition of progressive loss of T cell function, which was initially reported as clonal deletion of virus-specific T cells during high grade chronic CMV infections [15,16]. Gradual improvements in methodology for assessing T cell exhaustion identified that there was no clonal deletion of T cells but rather the T cells became unable to respond to stimuli due to sustained expression of inhibitory receptors that prevent effector function of T cells [15,16]. In addition to CMV infections, T cell exhaustion has also been reported following chronic exposure to HIV and/or Hepatitis C [17], Hepatitis B [18], *Plasmodium falciparum*[19] and *Toxoplasma gondii* (reviewed in [20]), but we have no evidence to suggest that our patient has been chronically infected by any of these pathogens. To the best of our knowledge, T cell exhaustion has not been documented in CF.

### Confirmation of T cell exhaustion

**Figure 2:**
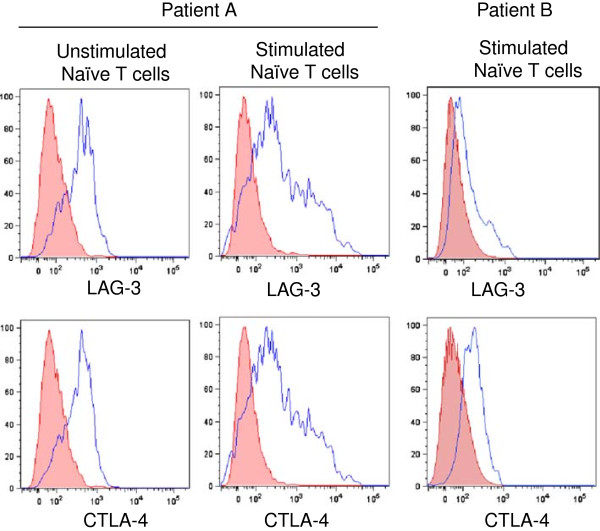
**Naïve T cells from Patient A, but not Patient B, showed marked induction of expression of the T cell exhaustion markers LAG-3 and CTLA-4 following stimulation with anti-CD3 and anti-CD28 antibodies.** Representative plots of the expression of the T cell exhaustion markers LAG-3 and CTLA-4 on unstimulated naïve T cells from patients A and B, and on T cells following stimulation with anti-CD3 and anti-CD28 antibodies. T cells from patient A, but not patient B, demonstrated a marked induction of the T cell exhaustion markers LAG-3 and CTLA-4. Representative of 2 - 3 independent experiments.

We hypothesized that the patient demonstrated T cell exhaustion, whereby even naïve T cells were inept at undergoing differentiation. In order to test this hypothesis, we assessed the levels of T cell exhaustion markers, LAG-3 [21] and CTLA-4 [16], on unstimulated as well as stimulated CD4+ T cells. Although LAG-3 and CTLA-4 are not expressed on naïve T cells from healthy individuals [22,23], freshly isolated naïve CD4+ T cells from peripheral blood of our present patient showed sustained expression of LAG-3 and CTLA-4, which were further induced even upon *ex vivo* stimulation with cytokines that drive T cell proliferation (Figure 2). These findings point towards T cell exhaustion as being the cause of the failure of the patient’s T cells to undergo differentiation in response to cytokine stimulation.

### Does the T cell exhaustion represent a primary or secondary immunodeficiency?

The patient’s respiratory secretions intermittently grew *P. aeruginosa*, among other common bacterial pathogens known for eliciting a vigorous immune response in CF. Given the sustained and severe lung inflammation that is typical of CF and given that chronic bacterial infections are essentially universal in end-stage CF lung disease, if such infection was at all likely to induce a secondary immunodeficiency, it would be expected that such secondary immunodeficiency would be commonly recognized in CF, which is not at all the case. Moreover, the subject of our present report had responses to measles, mumps, rubella and varicella-zoster vaccinations that appeared to be adequate, but remarkably little evidence of chronic viral infections potentially associated with immunosuppression. Therefore we suggest that the T cell exhaustion documented in our present patient likely represents a primary immunodeficiency, as a separate medical issue in addition to CF, and not a secondary outcome of ongoing CF pathology.

### Previous reports of T cell anomalies in CF

Circulating T lymphocytes are known to have defects in CFTR expression in CF [24]. Generally, reported abnormalities of T cells in CF have involved increased immune responses, especially relating to Th17 helper T cells, which have been linked to CF pulmonary exacerbations [9,25] and to neutrophilia very early in life [26]. Elevated sputum levels of Th17 cytokines are associated with active CF lung infections with *P. aeruginosa*. Elevated IL-17 levels predict future acquisition of *P. aeruginosa* infections [8]. Again, to the best of our knowledge T cell exhaustion has not been documented in CF.

### T cell exhaustion is *not* a universal occurrence in end-stage CF lung disease

We proceeded to also study another teen-aged girl with CF in our clinic, patient B, who had a very similar degree of lung dysfunction (e.g., FEV_1_ chronically < 30% of the predicted value) and who also underwent double lung transplantation since the initial submission of this report. Unlike the subject of our present report (patient A), whose T cells demonstrated a marked induction of the T cell exhaustion markers LAG-3 and CTLA-4, pre-transplant patient B showed only a mild induction of LAG-3 and CTLA-4 (Figure 2). Patient B also more closely resembled control than patient A, in that patient B showed Th1 and Th17 differentiation (Figures 1B, C), but compared to control showed somewhat more Th17 and less Th1, consistent with our recent report of an innate predisposition of naïve CF helper T cells to differentiate towards the Th17 phenotype [27]. Thus we cannot suggest that the T cell exhaustion described in patient A is an event that occurs in all patients at this stage of CF lung disease. It remains unknown if T cell exhaustion in CF is unique to patient A.

## Conclusions

In CF, chronic lung disease is the main cause of morbidity and mortality [3]. It is characterized by inflammation that is early, sustained and severe [4]. Although excessive inflammation in CF lung can be ineffective at clearing certain pathogens as well as destructive to the lung tissue itself [5], adequate inflammation is a component of an effective overall immune response to microbial pathogens. Our present findings suggest that intrinsic, primary impairment of T cell differentiation may have contributed to the overall greater severity and more rapid progression of our patient’s CF lung disease than is typical of the vast majority of her peers. An alternative explanation, that we consider less likely, is that her T cell exhaustion may be the late result of the severity of her CF lung disease. The prevalence and timing of T cell exhaustion in the general CF population is unknown; however, testing of another girl with end-stage CF lung disease did not detect any evidence of T cell exhaustion. Clarification of these issues may provide opportunities to design improved immunomodulatory treatment for CF.

## Consent

Written consent was obtained from the patient for the conduct of the investigation and the publication of this Case report. A copy of the written consent is available for review by the Editor-in-Chief of this journal.

## Abbreviations

CF: Cystic fibrosis; CFTR: CF transmembrane conductance regulator; CMV: Cytomegalovirus; FEV1: Forced expiratory volume, one second; Tregs: Regulatory T cells; Th1: T helper cells type 1; Th17: T helper cells type 17.

## Competing interests

The authors declare that they have no competing interests.

## Authors’ contributions

RK designed and conducted the immunological analysis of the T cell responses in the patient’s peripheral blood, collecting and interpreting data, and drafted the related portions of the paper. SG made substantial contributions to the acquisition of data. NBS drafted the abstract, background and clinical parts of the paper. All authors were involved in revising the manuscript critically for important intellectual content, and read and approved the final manuscript.
